# Large Language Model–Based Critical Care Big Data Deployment and Extraction: Descriptive Analysis

**DOI:** 10.2196/63216

**Published:** 2025-03-12

**Authors:** Zhongbao Yang, Shan-Shan Xu, Xiaozhu Liu, Ningyuan Xu, Yuqing Chen, Shuya Wang, Ming-Yue Miao, Mengxue Hou, Shuai Liu, Yi-Min Zhou, Jian-Xin Zhou, Linlin Zhang

**Affiliations:** 1 Department of Critical Care Medicine, Beijing Shijitan Hospital, Capital Medical University, Beijing, China; 2 School of Information Science and Technology, Beijing University of Technology, Beijing, China; 3 Department of Critical Care Medicine, Beijing Tiantan Hospital, Capital Medical University, No.119 Nansihuanxi Road, Fengtai District, Beijing, 100070, China, 86 17611757717

**Keywords:** big data, critical care–related databases, database deployment, large language model, database extraction, intensive care unit, ICU, GPT, artificial intelligence, AI, LLM

## Abstract

**Background:**

Publicly accessible critical care–related databases contain enormous clinical data, but their utilization often requires advanced programming skills. The growing complexity of large databases and unstructured data presents challenges for clinicians who need programming or data analysis expertise to utilize these systems directly.

**Objective:**

This study aims to simplify critical care–related database deployment and extraction via large language models.

**Methods:**

The development of this platform was a 2-step process. First, we enabled automated database deployment using Docker container technology, with incorporated web-based analytics interfaces Metabase and Superset. Second, we developed the intensive care unit–generative pretrained transformer (ICU-GPT), a large language model fine-tuned on intensive care unit (ICU) data that integrated LangChain and Microsoft AutoGen.

**Results:**

The automated deployment platform was designed with user-friendliness in mind, enabling clinicians to deploy 1 or multiple databases in local, cloud, or remote environments without the need for manual setup. After successfully overcoming GPT’s token limit and supporting multischema data, ICU-GPT could generate Structured Query Language (SQL) queries and extract insights from ICU datasets based on request input. A front-end user interface was developed for clinicians to achieve code-free SQL generation on the web-based client.

**Conclusions:**

By harnessing the power of our automated deployment platform and ICU-GPT model, clinicians are empowered to easily visualize, extract, and arrange critical care–related databases more efficiently and flexibly than manual methods. Our research could decrease the time and effort spent on complex bioinformatics methods and advance clinical research.

## Introduction

Critical care medicine has experienced an explosion of complex, heterogeneous data from various sources, such as electronic medical records, monitoring systems, imaging, registries, and omics platforms. This massive amount of multimodal data, often referred to as “big data,” offers enormous potential for improving patient outcomes in intensive care units (ICUs) through studies and analytics.

There are many high-quality medical databases that excel in the dimensions of volume, velocity, veracity, variety, and value. Examples include the Medical Information Mart for Intensive Care (MIMIC; MIMIC-III, MIMIC-IV, MIMIC-IV-Note, etc) at the Beth Israel Deaconess Medical Center in Boston [1-3], the Electronic Intensive Care Unit Collaborative Research Database (eICU-CRD) from 208 hospitals in the United States, and the Amsterdam University Medical Center Database (Amsterdam UMCdb) [4-6]. By integrating demographic, monitoring, laboratory, imaging, pharmacy, and waveform data, these databases provide a wealth of valuable information that has facilitated clinical research [7] and expanded the evidence base for clinical practice [8]. Many studies have demonstrated the potential of leveraging ICU big data for applications such as risk stratification, predictive models, treatment recommendations, and cohort phenotyping [9-12].

However, there are significant barriers to translating big data resources into clinical insights and decision support for frontline critical care teams. The complexity of ICU data requires specialized skills in data wrangling, mining, visualization, and interpretation, which most clinicians need to acquire. The gap between data science and clinical science hinders harnessing big data to improve ICU care [13,14]. Despite the promise of rich data and analytics, the adoption of actionable insights depends on accessible interfaces and informative data presentations [15].

Recent advances in medical natural language processing using artificial intelligence (AI) and large language models (LLMs) like GPTs have demonstrated the potential for intuitive human-computer interaction with health care data. Clinicians do not have to learn complex programming languages, and interfaces based on natural language and clinical logic can make big data analysis more accessible. An increasing body of research demonstrates that AI applications significantly enhance the efficiency and accuracy of disease diagnosis and treatment. Farič et al [16] developed an AI-based imaging software tool for detecting pulmonary nodules in chest computed tomography scans. Through qualitative semistructured interviews, they demonstrated the tool’s usability and effectiveness as both a decision-support system and a “second reader” for clinical users. Sjoding et al [17] employed a deep convolutional neural network to identify acute respiratory distress syndrome findings on chest radiographs. The convolutional neural network demonstrated expert physician-level accuracy in acute respiratory distress syndrome radiograph detection, as validated through comprehensive quantitative assessments using both internal and external test sets. However, general domain language models still face great challenges in handling niche biomedical data and require customization and continuous iterative improvement.

Therefore, we seek to empower clinicians by simplifying database deployment and developing the intensive care unit–generative pretrained transformer (ICU-GPT). This tool aims to simplify data extraction via LLMs, liberating clinicians from complex bioinformatics methods to focus on clinical research and data analysis. With ICU-GPT, clinicians can focus on clinical research advancement and feel more capable and in control of their data analysis.

## Methods

### Overview

To achieve the availability and visualization of critical care–related databases and simplify the process of data extraction, we developed this platform in 2 steps. Step 1 was database deployment based on Docker container technology through the web-based client rather than by installing other applications. Step 2 was the development of ICU-GPT based on LLMs to generate Structured Query Language (SQL) queries for data extraction after request input.

### Technologies of the Database Deployment and Visualization

Docker is an open-source platform that, by utilizing containerization technology, enables users to package the applications together with all their dependencies into an isolated container to achieve portability, consistency, and high segregation [18]. Database deployment involves the installation and configuration of health care databases onto PostgreSQL. This process ensures proper data storage and operational functionality. Based on Docker technology and customized PostgreSQL database initialization scripts [19], we aimed for a cross-platform automated deployment of critical care–related databases on different operating systems.

Database visualization leverages business intelligence tools to graphically represent the structure, content, and relationships within a database. This approach not only facilitates simple plotting but also provides a comprehensive and intuitive understanding of the database’s table structure and overall architecture. We provide 2 web-based clients with business intelligence tools to achieve database visualization: Metabase and Superset. Metabase and Superset are open-source data analytics and visualization tools for easy data exploration [20,21]. With simple and convenient working interfaces, they allow users to extract data from various sources without writing SQL statements, including relational databases (MySQL, PostgreSQL, SQL Server, etc) and NoSQL databases. They also enable users to add plugins and visualization components to meet specific requests with high extensibility and customizability.

### Technologies of ICU-GPT

With the appearance of the OpenAI GPT-3.5 in 2023, we decided to use LLMs to participate in the SQL query generation process [22]. An LLM is a natural language processing tool driven by AI technology and has recently emerged as a powerful tool across many areas of biomedicine. It can generate answers based on patterns observed during the pretraining stage, summarize large amounts of text, generate high-quality text from a short description, create code that can help support data analysis, produce images based on a verbal description, and much more.

ICU-GPT does not develop new LLM models but utilizes and is compatible with all OpenAI application programming interface (API) models. We chose OpenAI API-compatible models for ICU-GPT development for several reasons. First, our research team is more familiar with OpenAI. There is also a large number of models compatible with the OpenAI API, including GPT-3.5, GPT-4o, and GPT-4o mini, offering wide applications and cost-effectiveness. Users can select the appropriate OpenAI API model based on their specific requirements. Moreover, OpenAI encompasses knowledge bases from the MIMIC databases, which other models lack, including *International Classification of Diseases* (*ICD*) codes and itemid data.

Using the BIRD and Spider, 2 extensive cross-domain datasets designed to evaluate the impact of comprehensive database content on text-to-SQL parsing, we conducted a comparative analysis of OpenAI API models with other models [23,24]. The OpenAI API models demonstrated consistently high performance in execution accuracy and reward-based valid efficiency within the BIRD dataset [25]. Furthermore, these models exhibited stable and superior performance in execution with values within the Spider dataset [26]. Although reports suggest that certain models may surpass OpenAI’s performance, we were unable to include them in our comparison due to limitations in accessibility [27-30]. Moreover, due to the absence of a benchmark dataset specifically tailored for critical care–related databases, it remains challenging to directly compare ICU-GPT’s performance with other models on such databases.

Ollama is a lightweight framework with high extensibility for building and running LLMs on local and cloud environments [31]. It provides an API for model creation, execution, and management as well as a prebuilt model library. It is compatible with the OpenAI API for dialogue creation. The development of ICU-GPT also involved 4 technologies: LangChain, Microsoft AutoGen, Pandas, and Gradio.

LangChain is an open-source Python library that provides the modules and tools to build AI applications based on LLMs [32]. It can easily integrate with LLMs to achieve text generation, question-answering, and translation. The LangChain SQL database object was adopted and adjusted in our platform to support databases with multiple schemas, such as MIMIC and eICU-CRD.

Microsoft AutoGen is a framework that can be customized and conversed with, facilitating the development of LLM applications and seamlessly allowing for human participation [33]. Userproxy is a specialized agent within Microsoft AutoGen that receives users’ requests and generates appropriate prompts. GroupChat comprises 2 agents and Userproxy, working in tandem to facilitate the contemplation, execution, and refinement of users’ requests.

Pandas is a powerful Python-based library. It could provide accessible data structures to make data cleaning, transformation, and analysis more convenient [34]. Current data extraction functions are developed based on Pandas to achieve data processing.

Gradio is an open-source Python library. It enables developers to transform machine learning models and various functions into shareable web applications, facilitating a quick and easy showcasing of their work through intuitive and accessible interfaces [35].

## Results

**Figure 1.**
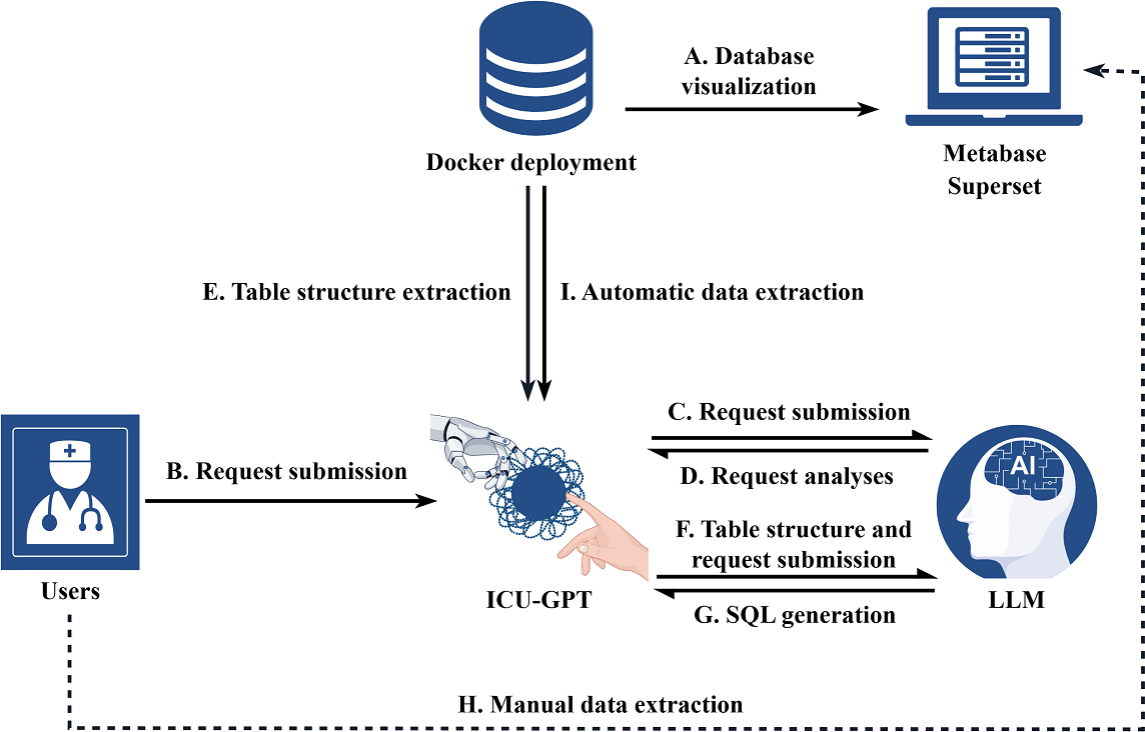
The procedures for the platform construction. The platform construction workflow encompasses several key components: database deployment based on Docker container technology; visualization powered by Metabase and Superset (A); SQL generation involving multiple rounds of interaction between users, LLMs, and ICU-GPT, including request submission, analysis, table structure extraction, and SQL generation (B to G); and data extraction (H and I). (A) Critical care–related database visualization based on Metabase and Superset. (B) Users send data extraction requests to ICU-GPT. (C) ICU-GPT sends requests to the LLM. (D) The LLM splits and analyzes the requests and sends them back to ICU-GPT. (E) ICU-GPT extracts table structure information from databases. (F) ICU-GPT sends requests and table structure information to the LLM. (G) The LLM generates SQL queries and sends them to ICU-GPT. (H) Users can choose to manually verify the SQL queries in Metabase and Superset. (I) ICU-GPT runs the SQL automatically for data extraction. ICU-GPT: intensive care unit–generative pretrained transformer; LLM: large language model; SQL: Structured Query Language. Figure created using Figdraw.

We summarized the workflow of database deployment, visualization, SQL generation, and data extraction in Figure 1.

### Database Deployment and Visualization

We adopted Docker technology to achieve the simplicity and flexibility of the cross-platform automated deployment of critical care–related databases. Functions of docker_process_init_files in docker-entrypoint.sh possess the mechanism of scanning shell files automatically in specific directories during the initial PostgreSQL container startup. The corresponding database initialization scripts are placed in the scanning path. Based on the mechanism, we named the files according to specific numbers to ensure the initialization process ran in the intended order and initialized the databases accordingly.

**Figure 2.**
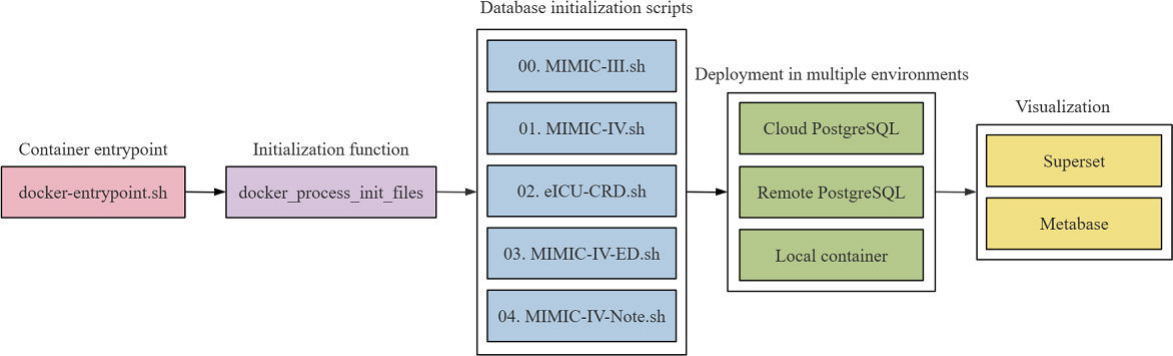
Deployment and visualization of critical care–related databases. eICU-CRD: Electronic Intensive Care Unit Collaborative Research Database; MIMIC: Medical Information Mart for Intensive Care; MIMIC-IV-ED: Medical Information Mart for Intensive Care IV – Emergency Department.

Our deployment platform supports several public critical care–related databases through PostgreSQL database initialization scripts, including MIMIC-III, MIMIC-IV, MIMIC-IV-ED (Medical Information Mart for Intensive Care IV – Emergency Department), MIMIC-IV-Note, eICU-CRD, etc. Clinicians can select the corresponding database initialization scripts or combine multiple scripts according to their specific requests. This design also allows clinicians to initialize the database with multiple phases in local, cloud, or remote environments and support database upgrades with great flexibility. Through 2 business intelligence solutions, Metabase and Superset, data querying, extraction, and charting can be easily performed by the web-based client without the installation of any other applications (Figure 2).

### SQL Query Generation and Data Extraction

We developed ICU-GPT to accurately assist in SQL query writing and provide a more intelligent and efficient approach to simplify data extraction.

Extensive research was conducted on SQL query generation. We found that most SQL query generation tools were only practical for tables under a single schema or merely supported a single table. The handling of databases with multiple schemas, such as MIMIC, was found to be poor. By thoroughly studying the source code of table information retrieval based on the LangChain SQL database object, we successfully overcame this deficiency and achieved multischema support.

Given the massive number of MIMIC tables, the actual tokens used to generate prompts exceed the upper limit of 16,000 tokens in OpenAI GPT-3.5. To address this issue, the table selection function was introduced to allow clinicians to generate SQL queries within a specific table range. This innovation not only solved the problem of the token limit but further improved the accuracy of SQL query generation. On the basis of this innovation, our platform is now highly flexible and available for more complex database structures.

Two agents and a Userproxy were established as a GroupChat using Microsoft AutoGen. One agent serves as the SQL engineer, responsible for SQL generation, while the other agent acts as the SQL expert, verifying and optimizing the SQL generated by the SQL engineer. A manager was also created to supervise the GroupChat and facilitate information interaction and agent selection within the group.

The Userproxy generates prompts based on clinicians’ requests and tables specified by users, then sends them to the manager. The manager transmits these prompts to agents and selects an appropriate agent to process the prompt using a relevant algorithm. This selected agent then sends the prompts to the LLM. The LLM generates SQL based on prompts and requests and then sends it back to the manager. The manager repeats this process, sending messages to agents and selecting the appropriate agent using the algorithm. The LLM receives prompts from the selected agent and interacts with the requests. The SQL expert evaluates the performance and accuracy of the generated SQL. If the SQL expert determines that the SQL accuracy is suboptimal, an iterative dialogue process is initiated to refine and improve the SQL as much as possible.

**Figure 3.**
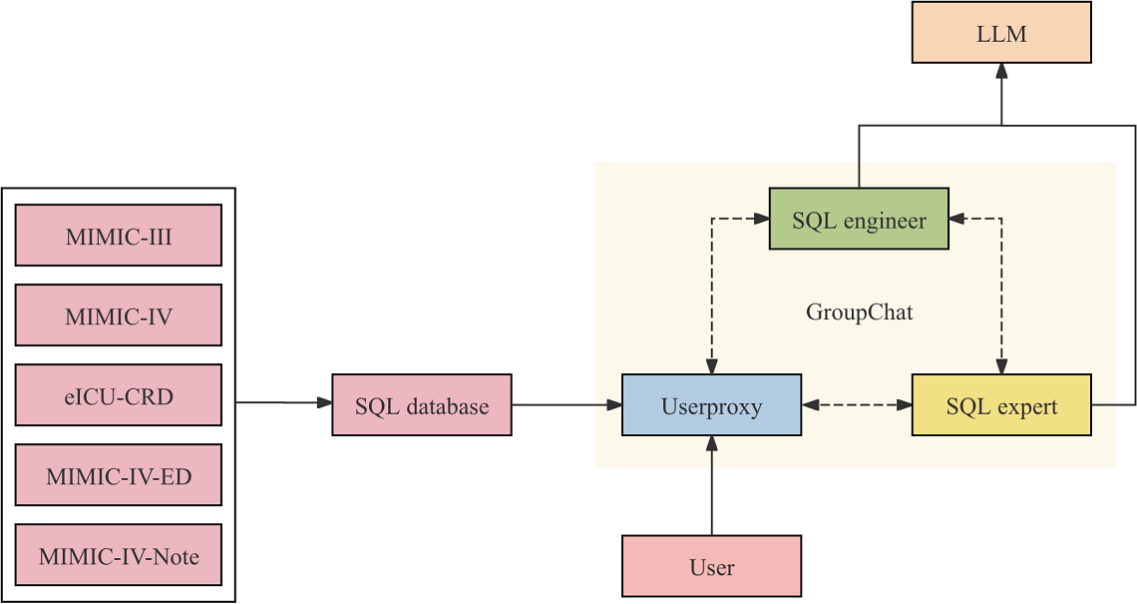
SQL query generation process. The GroupChat consists of the Userproxy, the SQL engineer, and the SQL expert. The manager is created to facilitate communication and information synchronization within the group. The SQL engineer is responsible for SQL generation and interaction with the LLM while the SQL expert is responsible for SQL optimization. The Userproxy generates prompts based on clinicians’ requests and user-specified tables. The manager then selects appropriate agents to process these prompts using the LLM. The LLM generates SQL queries, which are subsequently evaluated by the SQL expert. If necessary, an iterative dialogue ensues to refine the SQL for optimal accuracy and effectiveness. eICU-CRD: Electronic Intensive Care Unit Collaborative Research Database; LLM: large language model; MIMIC: Medical Information Mart for Intensive Care; MIMIC-IV-ED: Medical Information Mart for Intensive Care IV – Emergency Department; SQL: Structured Query Language.

As AI cannot fully replace human intelligence, there are limitations when users input prompts based on their research objectives. This is particularly evident when the entered keywords are not comprehensive, synonyms or similar prompts are used, or even when typos occur. In such cases, ICU-GPT may struggle to fully comprehend the user’s intent, potentially leading to incorrect or incomplete responses. To address this, SQL is not automatically executed after generation. Instead, users are given the opportunity to review and inspect the generated SQL themselves. If the results are unsatisfactory, users can further refine and optimize their prompts. This approach fosters a dynamic human-AI collaboration, maximizing SQL accuracy and user satisfaction (Figure 3) [36,37].

The code of the platform development is available online [38]. We warmly welcome and greatly appreciate any user feedback or new ideas, which can be submitted to us via GitHub. We will update ICU-GPT versions when the MIMIC and eICU-CRD databases are updated or when improved models become available. Some examples of how to achieve database deployment and SQL generation based on Python are presented in Multimedia Appendix 1.

### User Interface of ICU-GPT

**Figure 4.**
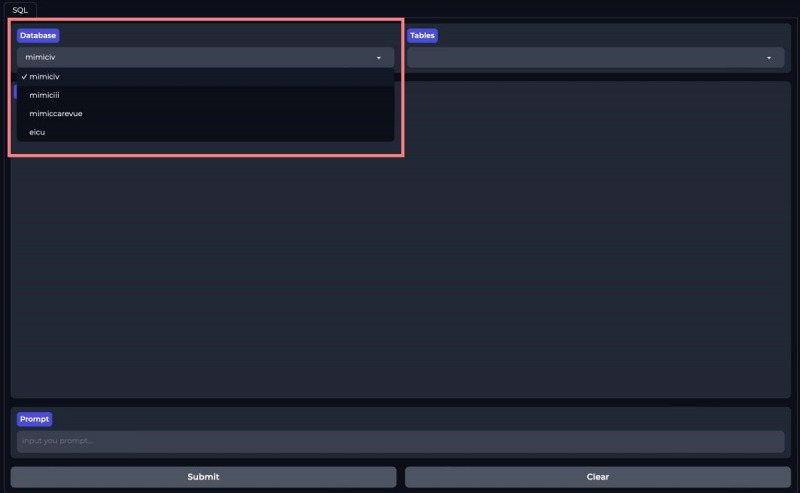
Users select databases on the ICU-GPT platform. ICU-GPT: intensive care unit–generative pretrained transformer.

**Figure 5.**
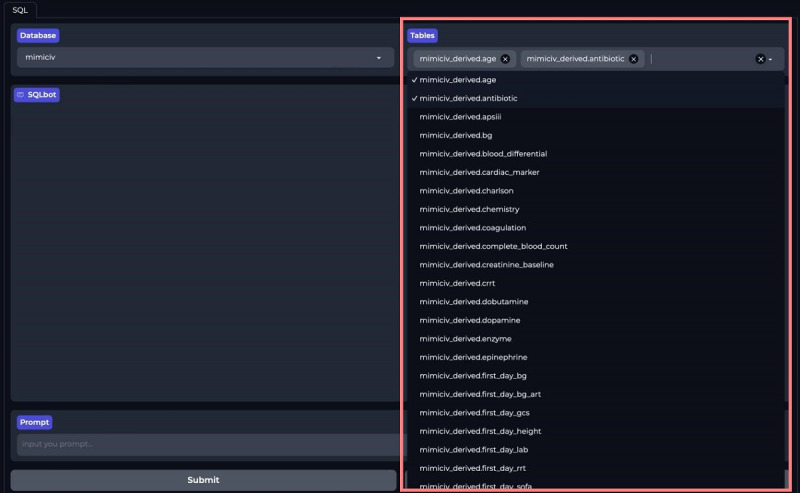
Users select tables on the ICU-GPT platform. ICU-GPT: intensive care unit–generative pretrained transformer.

**Figure 6.**
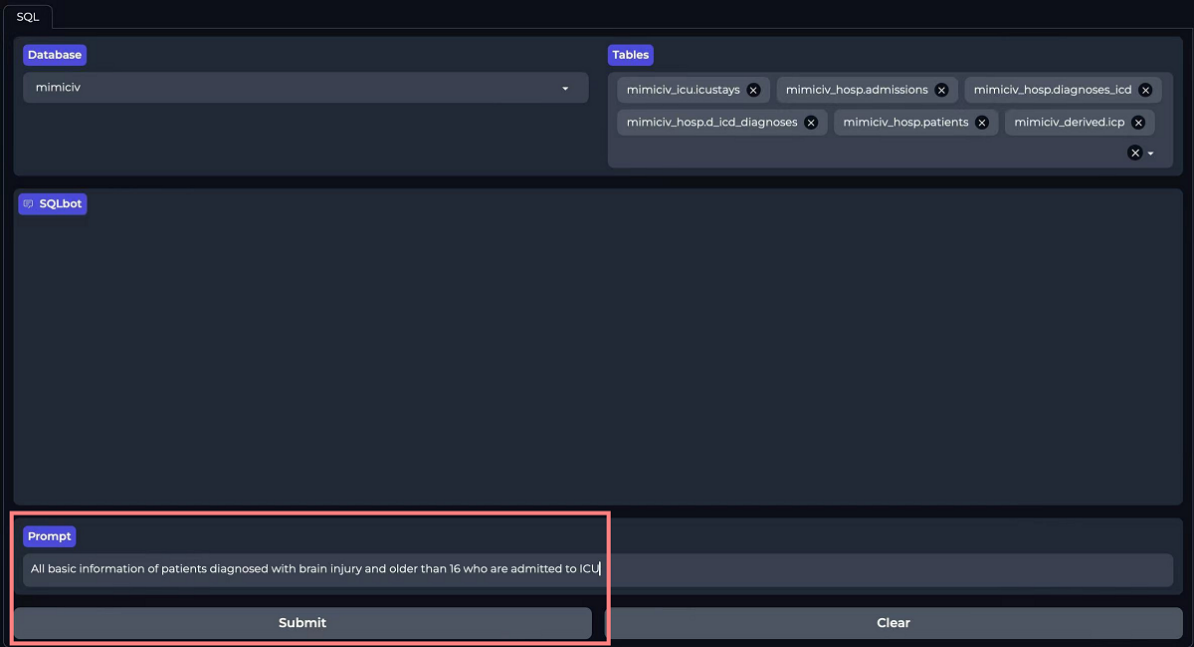
Users input and submit requests on the ICU-GPT platform. ICU-GPT: intensive care unit–generative pretrained transformer.

**Figure 7.**
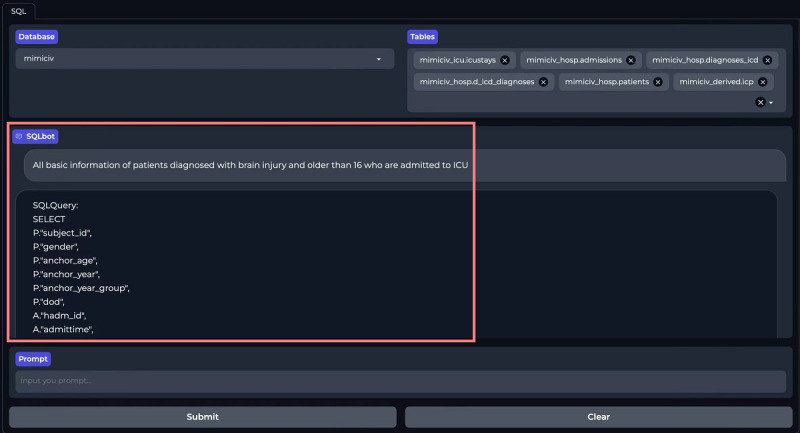
SQL output on the ICU-GPT platform. ICU-GPT: intensive care unit–generative pretrained transformer; SQL: Structured Query Language.

We developed a front-end user interface using Gradio and ICU-GPT to simplify the SQL generation process. Users can download our prebuilt Docker mirroring in the Dockerfile and follow the instructions to achieve code-free SQL generation on the web-based client [39]. First, users select a database: MIMIC-III, MIMIC-IV, MIMIC-CareVue, or eICU-CRD (Figure 4). The platform displays all table names under all schemas of the selected database. Users select the corresponding tables based on their requests (Figure 5). Users then input requests in the “Prompt” section and press “Submit” (Figure 6). Our platform supports prompts both in Chinese and English. Finally, the SQL bot outputs the SQL queries (Figure 7).

## Discussion

### Principal Results

In order to conduct the critical care–related database mining as efficiently and thoroughly as possible, we developed a platform in two steps: (1) we first implemented critical care–related database deployment by applying Docker container technology with 2 visualization tools, Metabase and Superset; (2) ICU-GPT was then developed based on LLMs to achieve SQL query generation and data extraction. By fully utilizing the advantages of open-source technology and being highly replicable and scalable, this platform provides great convenience for clinicians.

The critical care–related public databases cover a large sample size with various aspects during hospitalization, including demographic, physiologic, laboratory measurements, diagnoses, and medication administration data, which represents a certain degree of universality. While bringing tremendous clinical value, it is accompanied by the increasing complexity of database size and table structures. Even professional database administrators find it challenging to explore the table structures. Much time and effort would be spent on clinicians understanding the table structure and performing data extraction. Therefore, we simplified the complex and time-consuming process into 2 steps.

With Docker container technology, we first provided an efficient and reliable deployment solution to support the widespread application of critical care–related databases. With high repeatability and consistency, the automated deployment solution provides a professional and convenient database management and visualization tool for clinicians to arrange massive amounts of data.

After database deployment, we aimed to simplify the data extraction process. The Python peewee Object Relational Mapper library was considered for analyzing the database table structures automatically, binding them to peewee objects, and encapsulating the corresponding data extraction functions [40]. After multiple tests, although this approach could save the learning cost of SQL and database structures, programming foundation and data analysis experience in personalized extraction and data processing were still required. Despite extensive consideration of potential solutions for this problem, we could not achieve satisfactory outcomes, which led to a stagnation of the development process.

LLMs have recently emerged as powerful tools across many areas of biomedicine. As mentioned, they can rapidly summarize large amounts of text, generate high-quality text from a short description, create code that can help support data analysis, produce images based on a verbal description, and much more. Therefore, based on LLMs, we integrated LangChain and Microsoft AutoGen to develop the ICU-GPT. It not only overcame the token limit of GPT and supported multischema data, but it also achieved the seamless integration of SQL query generation and data extraction.

ICU-GPT empowers users with clinical expertise without data extraction proficiency to extract information from professional databases, significantly reducing the time spent on self-directed learning. However, quantifying the time saved and the efficiency gains from this learning process is complex, making it difficult to compare pre- and postimplementation efficiency improvements.

We agree that a comprehensive evaluation is crucial to demonstrate the system’s effectiveness and reliability. While this study focuses on design and implementation, we are actively implementing a thorough evaluation of ICU-GPT’s performance metrics. These include SQL query accuracy, system speed, user satisfaction, and the handling of large-scale datasets.

### Limitations and Implications

Our study has several limitations. First, our platform only involved MIMIC-III, MIMIC-IV, and eICU-CRD to demonstrate the methodological feasibility and clinical applicability of ICU-GPT in database deployment and data extraction in critical care–related databases. We will expand the research achievements to other databases and enrich the prompt templates of ICU-GPT, such as the Amsterdam UMCdb and high time–resolution intensive care unit dataset (HiRID) [6,41]. Issues of data security and ethical procedures require further improvement. The MIMIC and eICU-CRD databases were deidentified prior to their release, replacing identifiers with random integers while preserving data integrity to maximize data privacy to the fullest extent possible. However, more private databases may be developed in the future. Special attention should be paid particularly to these databases. Adherence to health care regulations, such as the Health Insurance Portability and Accountability Act (HIPAA) in the United States or the General Data Protection Regulation (GDPR) in Europe, is crucial. Second, multiple tests were conducted to verify the reliability and stability of the platform. Systematic evaluation and validation of this platform are warmly welcomed to facilitate further exploration of critical care–related databases. Third, ICU-GPT mainly focuses on structured data SQL generation and analysis. We plan to leverage function calls and the Medical Concept Annotation Toolkit (MedCAT) to achieve unstructured data extraction and analysis like clinical notes from electronic health records in the future [42]. This will enable us to link the unstructured data to biomedical models, facilitating more comprehensive database mining. We will continue to enhance ICU-GPT and strive to achieve automated data processing through Pandas. Fourth, ICU-GPT, as a system built on existing LLMs, may inherit certain biases or limitations from the underlying models. These biases are a known challenge with LLMs due to the nature of their training on large-scale datasets, which may contain imbalances or inaccuracies. To mitigate these risks, we have implemented several measures. We chose OpenAI API compatible models for ICU-GPT development for its incorporation of knowledge bases from the MIMIC database, which includes crucial *ICD* codes and itemid data that other models lack. The system is designed to be used as a human-in-the-loop tool rather than an autonomous system, with users reviewing and validating the generated outputs. We also actively monitor the system’s performance in real-world applications and incorporate user feedback to iteratively improve accuracy and reliability. Moreover, periodic audits are conducted to identify and address potential biases in the system’s responses. Fifth, it is indispensable to note that AI cannot replace human intelligence and judgment. Cautious inspection and correction are required for platform improvement.

Despite the aforementioned limitations, this study addresses the development of ICU-GPT to simplify critical care–related database deployment and extraction via LLMs. Further work may focus on enhancing the intelligence level of ICU-GPT, such as achieving autonomous SQL generation based on complex requests, rather than relying solely on fixed templates. It is also anticipated that researchers and developers will create advanced tools for the auxiliary diagnosis and treatment of critical illnesses, as well as sophisticated diagnostic decision support systems based on ICU-GPT.

**Figure 8.**
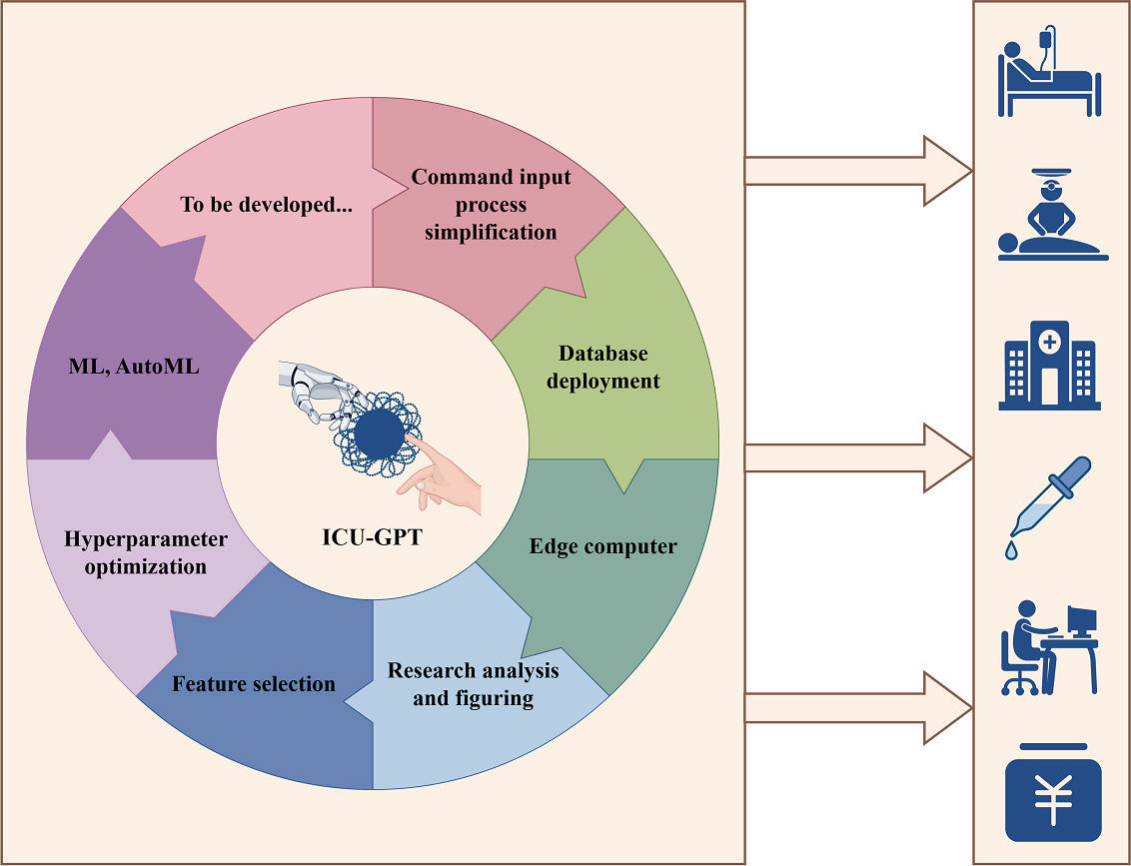
Expansion of ICU-GPT functions and its potential benefits. ICU-GPT will bring significant benefits after the expansion of its functions, enhancing patient care, clinical work, hospital development, academic progress, administrative management, and business improvement. AutoML: automated machine learning; ICU-GPT: intensive care unit–generative pretrained transformer; ML: machine learning. Figure created using Figdraw.

The massive expansion of critical care–related databases and functions in ICU-GPT will offer enormous potential to uncover patterns, derive predictive models, tailor interventions, and enable precision medicine through data science [43]. This will further hold immense potential and promising broad prospects for patient care, clinical work, hospital development, academic progress, administrative management, and business improvement (Figure 8). This area of research is ripe for exploration and could lead to groundbreaking advancements in the field.

### Conclusions

Leveraging our automated deployment platform and ICU-GPT model, clinicians can now easily visualize, extract, and organize public critical care–related datasets with greater efficiency and flexibility compared to manual methods. By reducing the time and complexity of bioinformatics analysis, our platform and customized LLM enable clinicians without programming expertise to harness large ICU databases for clinical research advancements. The end-to-end solution from deployment to analysis makes complex data more accessible to frontline ICU staff, unlocking the potential of big data to enhance evidence-based care and outcomes for critically ill patients. Overall, this work demonstrates the promise of thoughtful human-AI collaboration in transforming critical care delivery through data-driven insights.

## Supplementary material

10.2196/63216 Multimedia Appendix 1 Case demonstration of the platform applicability based on Python.

## Abbreviations

AI: artificial intelligence
Amsterdam UMCdb: Amsterdam University Medical Center Database
API: application programming interface
eICU-CRD: Electronic Intensive Care Unit Collaborative Research Database
GDPR: General Data Protection Regulation
HIPAA: Health Insurance Portability and Accountability Act
HiRID: high time–resolution intensive care unit dataset
*ICD*: *International Classification of Diseases*
ICU: intensive care unit
ICU-GPT: intensive care unit–generative pretrained transformer
LLM: large language model
MedCAT: Medical Concept Annotation Toolkit
MIMIC: Medical Information Mart for Intensive Care
MIMIC-IV-ED: Medical Information Mart for Intensive Care IV – Emergency Department
ML: machine learning
SQL: Structured Query Language
