# Supplementary material for: Large Language Model–Based Critical Care Big Data Deployment and Extraction: Descriptive Analysis
Source: JMIR Med Inform. 2025 Mar 12;13:e63216. doi: 10.2196/63216 (PMC11922493; doi:10.2196/63216)
Supplement: Multimedia Appendix 1 [file medinform-v13-e63216-s001.docx]

Multimedia Appendix 1. Case demonstration of the platform applicability based on Python.

(A) Database deployment

(1) Change the data directory and copy the initialization bash to the directory you want.

*version: '3.9'*

*services:*

*metabase:*

*image: metabase/metabase:latest*

*container_name: metabase*

*hostname: metabase*

*volumes:*

*- /dev/urandom:/dev/random:ro*

*ports:*

*- 3000:3000*

*environment:*

*MB_DB_TYPE: postgres*

*MB_DB_DBNAME: metabase*

*MB_DB_PORT: 5432*

*MB_DB_USER: postgres*

*MB_DB_PASS: mimic*

*MB_DB_HOST: postgres*

*depends_on:*

*- postgres*

*postgres:*

*image: postgres:latest*

*command: >*

*-c maintenance_work_mem=256MB*

*-c max_wal_size=4GB*

*container_name: postgres*

*hostname: postgres*

*volumes:*

*- ${PWD}/data:/var/lib/postgresql/data ## Change ${PWD} into the directory where you want to store data*

*- ${PWD}/icu/:/docker-entrypoint-initdb.d/:ro ##* *Copy the initialization scripts to the directory where you want to store data, and change the ${PWD} to the actual directory*

*ports:*

*- 5432:5432*

*environment:*

*POSTGRES_USER: postgres*

*POSTGRES_PASSWORD: mimic*

(2) Database preparation

*#Download mimiciii ed mimic-note mimiciii-carevue mimiciv ... csv.gz data*

*# cp mimiciii/*csv.gz to ${PWD}/icu/data/mimiciii/*

*# cp ed/*csv.gz to ${PWD}/icu/data/ed/*

*# cp mimic-carevue/*csv.gz to ${PWD}/icu/data/mimic-carevue/*

*# cp eicu/*csv.gz to ${PWD}/icu/data/eicu/*

*# cp mimic-iv/*csv.gz to ${PWD}/icu/data/mimic-iv/*

(3) Database initialization

*chmod +x install*

*./install*

(4) Initialization check

*docker ps |grep postgres*

*docker ps |grep metabase*

*# or you can run install dird*

*# docker compose ps –all*

(5) Check logs

*docker logs postgres ## view postgres you can check db init progress*

(6) Restart Sevice

*# in the install dir*

*docker compose restart*

(7) Uninstall Sevice

*# in the install dir*

*docker compose down -v*

(B) SQL query generation

(1) Users can achieve SQL query generation and data extraction through ICU-GPT. ICU-GPT is installed in two ways.

First, Pip for ICU-GPT installation: [*https://github.com/ICU-GPT/icu-gpt.git*](https://github.com/ICU-GPT/icu-gpt.git)*.* Second, source code for ICU-GPT installation:

*git clone* [*https://github.com/ICU-GPT/icu-gpt*](https://github.com/ICU-GPT/icu-gpt)

*cd icu-gpt*

*pip install. ## or python steup.py install*

(2) After ICU-GPT installation, users need to prepare the OpenAI API key as well as the account and password of critical care-related databases and store them in .env file:

*OPENAI_API_KEY="sk-**" # your OpenAI API key*

*PG_USER = '…' # your database username*

*PG_PASSWD = '…' # your database password*

*PG_SERVER = '……' # your IP address*

(3) When you import icu-gpt, .env file will import automatically. LLM-configuration initialization for OpenAI client initialization:

*from autogen.oai import config_list_from_models*

*# build the gpt_configuration object*

*# Base Configuration*

*base_config = {*

*"temperature": 0,*

*"config_list": config_list_from_models(model_list=["gpt-4"]),*

*}*

(4) ICU-GPT initialization for SQL generation:

*import icugpt*

*ag = icugpt.ICU(db_name='…',pg_user='…',pg_passwd='…',pg_server='…',llm_config=base_config) ## replace databases names, username, password, and IP address with ellipsis.*

*ag.sql_chat(prompt='…',tables=["…"]) ## replace data extraction requests, patients diagnosis and table extraction range with ellipsis for.*

Remember to use the characters involved in the International Statistical Classification of Diseases and Related Health Problems (ICD) when entering the diagnosis.

(C) Example 1

If you want to extract all the information of brain-injured patients admitted into ICU with age older than 16, the code will be as follows:

*import icugpt*

*ag = icugpt.ICU(db_name='mimiciv',pg_user='postgres',pg_passwd='mimic',pg_server='…',llm_config=base_config)*

*ag.sql_chat(prompt='All basic information of patients diagnosed with brain injury and older than 16 who are admitted to ICU*

*]',tables=["mimiciv_icu.icustays","mimiciv_hosp.admissions","mimiciv_hosp.diagnoses_icd","mimiciv_hosp.d_icd_diagnoses", "mimiciv_hosp.patients","mimiciv_derived.icp"])*

After requests input, ICU-GPT analyses and output the corresponding prompts as follows:

*ICUagent (to chat_manager):*

*When answering an input question:Query only the necessary columns from the relevant tables. Avoid querying all columns unless absolutely required. Wrap column names in double quotes (") to denote delimited identifiers. Ensure all queried columns exist in the provided tables.*

*Question: All basic information of patients diagnosed with brain injury and older than 16 who are admitted to ICU*

*Only use the following tables:*

*CREATE TABLE mimiciv_derived.icp (*

*subject_id INTEGER,*

*stay_id INTEGER,*

*charttime TIMESTAMP WITHOUT TIME ZONE,*

*icp DOUBLE PRECISION*

*)*

*/**

*3 rows from icp table:*

*subject_id stay_id charttime icp*

*10025463 38275267 2137-10-09 02:54:00 36.0*

*10025463 38275267 2137-10-09 03:15:00 11.0*

*10025463 38275267 2137-10-09 04:00:00 11.0*

**/*

*CREATE TABLE mimiciv_hosp.d_icd_diagnoses (*

*icd_code CHAR(7) NOT NULL,*

*icd_version SMALLINT NOT NULL,*

*long_title VARCHAR(255),*

*CONSTRAINT d_icd_diagnoses_pk PRIMARY KEY (icd_code, icd_version)*

*)*

*/**

*3 rows from d_icd_diagnoses table:*

*icd_code icd_version long_title*

*0010 9 Cholera due to vibrio cholerae*

*0011 9 Cholera due to vibrio cholerae el tor*

*0019 9 Cholera, unspecified*

**/*

*CREATE TABLE mimiciv_hosp.patients (*

*subject_id INTEGER NOT NULL,*

*gender CHAR(1) NOT NULL,*

*anchor_age SMALLINT,*

*anchor_year SMALLINT NOT NULL,*

*anchor_year_group VARCHAR(20) NOT NULL,*

*dod DATE,*

*CONSTRAINT patients_pk PRIMARY KEY (subject_id)*

*)*

*/**

*3 rows from patients table:*

*subject_id gender anchor_age anchor_year anchor_year_group dod*

*10000032 F 52 2180 2014 - 2016 2180-09-09*

*10000048 F 23 2126 2008 - 2010 None*

*10000068 F 19 2160 2008 - 2010 None*

**/*

*CREATE TABLE mimiciv_hosp.admissions (*

*subject_id INTEGER NOT NULL,*

*hadm_id INTEGER NOT NULL,*

*admittime TIMESTAMP WITHOUT TIME ZONE NOT NULL,*

*dischtime TIMESTAMP WITHOUT TIME ZONE,*

*deathtime TIMESTAMP WITHOUT TIME ZONE,*

*admission_type VARCHAR(40) NOT NULL,*

*admit_provider_id VARCHAR(10),*

*admission_location VARCHAR(60),*

*discharge_location VARCHAR(60),*

*insurance VARCHAR(255),*

*language VARCHAR(10),*

*marital_status VARCHAR(30),*

*race VARCHAR(80),*

*edregtime TIMESTAMP WITHOUT TIME ZONE,*

*edouttime TIMESTAMP WITHOUT TIME ZONE,*

*hospital_expire_flag SMALLINT,*

*CONSTRAINT admissions_pk PRIMARY KEY (hadm_id),*

*CONSTRAINT admissions_patients_fk FOREIGN KEY(subject_id) REFERENCES mimiciv_hosp.patients (subject_id)*

*)*

*/**

*3 rows from admissions table:*

*subject_id hadm_id admittime dischtime deathtime admission_type admit_provider_id admission_location discharge_location insurance language marital_status race edregtime edouttime hospital_expire_flag*

*10000032 22595853 2180-05-06 22:23:00 2180-05-07 17:15:00 None URGENT P874LG TRANSFER FROM HOSPITAL HOME Other ENGLISH WIDOWED WHITE 2180-05-06 19:17:00 2180-05-06 23:30:00 0*

*10000032 22841357 2180-06-26 18:27:00 2180-06-27 18:49:00 None EW EMER. P09Q6Y EMERGENCY ROOM HOME Medicaid ENGLISH WIDOWED WHITE 2180-06-26 15:54:00 2180-06-26 21:31:00 0*

*10000032 25742920 2180-08-05 23:44:00 2180-08-07 17:50:00 None EW EMER. P60CC5 EMERGENCY ROOM HOSPICE Medicaid ENGLISH WIDOWED WHITE 2180-08-05 20:58:00 2180-08-06 01:44:00 0*

**/*

*CREATE TABLE mimiciv_hosp.diagnoses_icd (*

*subject_id INTEGER NOT NULL,*

*hadm_id INTEGER NOT NULL,*

*seq_num INTEGER NOT NULL,*

*icd_code CHAR(7) NOT NULL,*

*icd_version SMALLINT NOT NULL,*

*CONSTRAINT diagnoses_icd_pk PRIMARY KEY (hadm_id, seq_num, icd_code, icd_version),*

*CONSTRAINT diagnoses_icd_admissions_fk FOREIGN KEY(hadm_id) REFERENCES mimiciv_hosp.admissions (hadm_id),*

*CONSTRAINT diagnoses_icd_patients_fk FOREIGN KEY(subject_id) REFERENCES mimiciv_hosp.patients (subject_id)*

*)*

*/**

*3 rows from diagnoses_icd table:*

*subject_id hadm_id seq_num icd_code icd_version*

*10000032 22595853 1 5723 9*

*10000032 22595853 2 78959 9*

*10000032 22595853 3 5715 9*

**/*

*CREATE TABLE mimiciv_icu.icustays (*

*subject_id INTEGER NOT NULL,*

*hadm_id INTEGER NOT NULL,*

*stay_id INTEGER NOT NULL,*

*first_careunit VARCHAR(255),*

*last_careunit VARCHAR(255),*

*intime TIMESTAMP WITHOUT TIME ZONE,*

*outtime TIMESTAMP WITHOUT TIME ZONE,*

*los DOUBLE PRECISION,*

*CONSTRAINT icustays_pk PRIMARY KEY (stay_id),*

*CONSTRAINT icustays_admissions_fk FOREIGN KEY(hadm_id) REFERENCES mimiciv_hosp.admissions (hadm_id),*

*CONSTRAINT icustays_patients_fk FOREIGN KEY(subject_id) REFERENCES mimiciv_hosp.patients (subject_id)*

*)*

*/**

*3 rows from icustays table:*

*subject_id hadm_id stay_id first_careunit last_careunit intime outtime los*

*10000032 29079034 39553978 Medical Intensive Care Unit (MICU) Medical Intensive Care Unit (MICU) 2180-07-23 14:00:00 2180-07-23 23:50:47 0.4102662037037037*

*10000980 26913865 39765666 Medical Intensive Care Unit (MICU) Medical Intensive Care Unit (MICU) 2189-06-27 08:42:00 2189-06-27 20:38:27 0.4975347222222222*

*10001217 24597018 37067082 Surgical Intensive Care Unit (SICU) Surgical Intensive Care Unit (SICU) 2157-11-20 19:18:02 2157-11-21 22:08:00 1.1180324074074075*

**/*

*--------------------------------------------------------------------------------*

*Next speaker: sql-agent*

*sql-agent (to chat_manager):*

*```sql*

*-- SQL query to retrieve basic information of patients diagnosed with brain injury and older than 16 who are admitted to ICU*

*SELECT*

*p."subject_id",*

*p."gender",*

*p."anchor_age",*

*p."anchor_year",*

*a."admittime",*

*a."dischtime",*

*a."admission_type",*

*a."admission_location",*

*a."discharge_location",*

*a."insurance",*

*a."language",*

*a."marital_status",*

*a."race"*

*FROM*

*mimiciv_hosp.patients p*

*JOIN*

*mimiciv_hosp.admissions a ON p."subject_id" = a."subject_id"*

*JOIN*

*mimiciv_hosp.diagnoses_icd d ON a."hadm_id" = d."hadm_id"*

*JOIN*

*mimiciv_hosp.d_icd_diagnoses di ON d."icd_code" = di."icd_code" AND d."icd_version" = di."icd_version"*

*JOIN*

*mimiciv_icu.icustays icu ON a."hadm_id" = icu."hadm_id"*

*WHERE*

*di."long_title" ILIKE '%brain injury%' -- Assuming 'brain injury' is part of the diagnosis description*

*AND p."anchor_age" > 16;*

*```*

*This query retrieves the basic information of patients who have been diagnosed with a brain injury, are older than 16, and have been admitted to the ICU. It joins the necessary tables to filter based on the diagnosis and age criteria.*

*--------------------------------------------------------------------------------*

*Next speaker: expert_agent*

*expert_agent (to chat_manager):*

*The SQL query you've provided is well-structured and mostly adheres to best practices. However, there are a few points to consider for improvement:*

*1. **Diagnosis Filtering**: The use of `ILIKE '%brain injury%'` assumes that the term "brain injury" is part of the `long_title` in the `d_icd_diagnoses` table. Ensure that this assumption is correct. If the exact ICD codes for brain injuries are known, it would be more efficient to filter using those specific codes.*

*2. **Column Selection**: The query selects a comprehensive set of columns from the `patients` and `admissions` tables. Ensure that all these columns are necessary for the intended analysis. If not, consider selecting only the essential columns to improve query performance.*

*3. **Formatting and Readability**: The query is well-formatted and easy to read, which is good for maintainability.*

*4. **Use of Double Quotes**: The use of double quotes around column names is consistent and adheres to PostgreSQL's handling of delimited identifiers.*

*5. **Join Conditions**: The join conditions are correctly specified, ensuring that the relationships between tables are respected.*

*Overall, the query is well-constructed, but verifying the diagnosis filtering criteria and ensuring that only necessary columns are selected would enhance its efficiency and accuracy.*

*--------------------------------------------------------------------------------*

*Next speaker: ICUagent*

*ICUagent (to chat_manager):*

*--------------------------------------------------------------------------------*

*Next speaker: sql-agent*

*sql-agent (to chat_manager):*

*```sql*

*-- SQL query to retrieve basic information of patients diagnosed with brain injury and older than 16 who are admitted to ICU*

*SELECT*

*p."subject_id",*

*p."gender",*

*p."anchor_age",*

*p."anchor_year",*

*a."admittime",*

*a."dischtime",*

*a."admission_type",*

*a."admission_location",*

*a."discharge_location",*

*a."insurance",*

*a."language",*

*a."marital_status",*

*a."race"*

*FROM*

*mimiciv_hosp.patients p*

*JOIN*

*mimiciv_hosp.admissions a ON p."subject_id" = a."subject_id"*

*JOIN*

*mimiciv_hosp.diagnoses_icd d ON a."hadm_id" = d."hadm_id"*

*JOIN*

*mimiciv_hosp.d_icd_diagnoses di ON d."icd_code" = di."icd_code" AND d."icd_version" = di."icd_version"*

*JOIN*

*mimiciv_icu.icustays icu ON a."hadm_id" = icu."hadm_id"*

*WHERE*

*di."long_title" ILIKE '%brain injury%' -- Assuming 'brain injury' is part of the diagnosis description*

*AND p."anchor_age" > 16;*

*```*

*This query retrieves the basic information of patients who have been diagnosed with a brain injury, are older than 16, and have been admitted to the ICU. It joins the necessary tables to filter based on the diagnosis and age criteria. Ensure that the diagnosis filtering is accurate by confirming the presence of "brain injury" in the `long_title` or by using specific ICD codes if available.*

*--------------------------------------------------------------------------------*

*Next speaker: expert_agent*

*expert_agent (to chat_manager):*

*The SQL query you've provided is well-structured and adheres to PostgreSQL best practices. It effectively joins the necessary tables and applies the correct filters to retrieve the desired information. The use of double quotes for column names is consistent, and the query is formatted for readability.*

*The only suggestion is to ensure that the diagnosis filtering using `ILIKE '%brain injury%'` accurately captures all relevant cases. If specific ICD codes for brain injuries are known, using those codes would be more precise and efficient.*

*Overall, the query is well-constructed and should produce accurate results given the assumptions about the diagnosis filtering. BINGO!*

*--------------------------------------------------------------------------------*

*Next speaker: ICUagent*

(D) Example 2

Our platform also supports the input of Chinese prompts. But for diagnosis in the prompts, we suggest inputting the English characters involved in ICD, as errors may arise when using other languages.

*ag.sql_chat(prompt='诊断为brain injury且年龄大于16的 入住icu 病人所有基础信息',tables=["mimiciv_icu.icustays","mimiciv_hosp.admissions","mimiciv_hosp.diagnoses_icd","mimiciv_hosp.d_icd_diagnoses", "mimiciv_hosp.patients","mimiciv_derived.icp"]) ## Start sql generation*

After requests input, ICU-GPT analyses and output the corresponding prompts as follows:

*ICUagent (to chat_manager):*

*When answering an input question:Query only the necessary columns from the relevant tables. Avoid querying all columns unless absolutely required. Wrap column names in double quotes (") to denote delimited identifiers. Ensure all queried columns exist in the provided tables.*

*Question: 诊断为brain injury且年龄大于16的 入住icu 病人所有基础信息*

*Only use the following tables:*

*CREATE TABLE mimiciv_derived.icp (*

*subject_id INTEGER,*

*stay_id INTEGER,*

*charttime TIMESTAMP WITHOUT TIME ZONE,*

*icp DOUBLE PRECISION*

*)*

*/**

*3 rows from icp table:*

*subject_id stay_id charttime icp*

*10025463 38275267 2137-10-09 02:54:00 36.0*

*10025463 38275267 2137-10-09 03:15:00 11.0*

*10025463 38275267 2137-10-09 04:00:00 11.0*

**/*

*CREATE TABLE mimiciv_hosp.d_icd_diagnoses (*

*icd_code CHAR(7) NOT NULL,*

*icd_version SMALLINT NOT NULL,*

*long_title VARCHAR(255),*

*CONSTRAINT d_icd_diagnoses_pk PRIMARY KEY (icd_code, icd_version)*

*)*

*/**

*3 rows from d_icd_diagnoses table:*

*icd_code icd_version long_title*

*0010 9 Cholera due to vibrio cholerae*

*0011 9 Cholera due to vibrio cholerae el tor*

*0019 9 Cholera, unspecified*

**/*

*CREATE TABLE mimiciv_hosp.patients (*

*subject_id INTEGER NOT NULL,*

*gender CHAR(1) NOT NULL,*

*anchor_age SMALLINT,*

*anchor_year SMALLINT NOT NULL,*

*anchor_year_group VARCHAR(20) NOT NULL,*

*dod DATE,*

*CONSTRAINT patients_pk PRIMARY KEY (subject_id)*

*)*

*/**

*3 rows from patients table:*

*subject_id gender anchor_age anchor_year anchor_year_group dod*

*10000032 F 52 2180 2014 - 2016 2180-09-09*

*10000048 F 23 2126 2008 - 2010 None*

*10000068 F 19 2160 2008 - 2010 None*

**/*

*CREATE TABLE mimiciv_hosp.admissions (*

*subject_id INTEGER NOT NULL,*

*hadm_id INTEGER NOT NULL,*

*admittime TIMESTAMP WITHOUT TIME ZONE NOT NULL,*

*dischtime TIMESTAMP WITHOUT TIME ZONE,*

*deathtime TIMESTAMP WITHOUT TIME ZONE,*

*admission_type VARCHAR(40) NOT NULL,*

*admit_provider_id VARCHAR(10),*

*admission_location VARCHAR(60),*

*discharge_location VARCHAR(60),*

*insurance VARCHAR(255),*

*language VARCHAR(10),*

*marital_status VARCHAR(30),*

*race VARCHAR(80),*

*edregtime TIMESTAMP WITHOUT TIME ZONE,*

*edouttime TIMESTAMP WITHOUT TIME ZONE,*

*hospital_expire_flag SMALLINT,*

*CONSTRAINT admissions_pk PRIMARY KEY (hadm_id),*

*CONSTRAINT admissions_patients_fk FOREIGN KEY(subject_id) REFERENCES mimiciv_hosp.patients (subject_id)*

*)*

*/**

*3 rows from admissions table:*

*subject_id hadm_id admittime dischtime deathtime admission_type admit_provider_id admission_location discharge_location insurance language marital_status race edregtime edouttime hospital_expire_flag*

*10000032 22595853 2180-05-06 22:23:00 2180-05-07 17:15:00 None URGENT P874LG TRANSFER FROM HOSPITAL HOME Other ENGLISH WIDOWED WHITE 2180-05-06 19:17:00 2180-05-06 23:30:00 0*

*10000032 22841357 2180-06-26 18:27:00 2180-06-27 18:49:00 None EW EMER. P09Q6Y EMERGENCY ROOM HOME Medicaid ENGLISH WIDOWED WHITE 2180-06-26 15:54:00 2180-06-26 21:31:00 0*

*10000032 25742920 2180-08-05 23:44:00 2180-08-07 17:50:00 None EW EMER. P60CC5 EMERGENCY ROOM HOSPICE Medicaid ENGLISH WIDOWED WHITE 2180-08-05 20:58:00 2180-08-06 01:44:00 0*

**/*

*CREATE TABLE mimiciv_hosp.diagnoses_icd (*

*subject_id INTEGER NOT NULL,*

*hadm_id INTEGER NOT NULL,*

*seq_num INTEGER NOT NULL,*

*icd_code CHAR(7) NOT NULL,*

*icd_version SMALLINT NOT NULL,*

*CONSTRAINT diagnoses_icd_pk PRIMARY KEY (hadm_id, seq_num, icd_code, icd_version),*

*CONSTRAINT diagnoses_icd_admissions_fk FOREIGN KEY(hadm_id) REFERENCES mimiciv_hosp.admissions (hadm_id),*

*CONSTRAINT diagnoses_icd_patients_fk FOREIGN KEY(subject_id) REFERENCES mimiciv_hosp.patients (subject_id)*

*)*

*/**

*3 rows from diagnoses_icd table:*

*subject_id hadm_id seq_num icd_code icd_version*

*10000032 22595853 1 5723 9*

*10000032 22595853 2 78959 9*

*10000032 22595853 3 5715 9*

**/*

*CREATE TABLE mimiciv_icu.icustays (*

*subject_id INTEGER NOT NULL,*

*hadm_id INTEGER NOT NULL,*

*stay_id INTEGER NOT NULL,*

*first_careunit VARCHAR(255),*

*last_careunit VARCHAR(255),*

*intime TIMESTAMP WITHOUT TIME ZONE,*

*outtime TIMESTAMP WITHOUT TIME ZONE,*

*los DOUBLE PRECISION,*

*CONSTRAINT icustays_pk PRIMARY KEY (stay_id),*

*CONSTRAINT icustays_admissions_fk FOREIGN KEY(hadm_id) REFERENCES mimiciv_hosp.admissions (hadm_id),*

*CONSTRAINT icustays_patients_fk FOREIGN KEY(subject_id) REFERENCES mimiciv_hosp.patients (subject_id)*

*)*

*/**

*3 rows from icustays table:*

*subject_id hadm_id stay_id first_careunit last_careunit intime outtime los*

*10000032 29079034 39553978 Medical Intensive Care Unit (MICU) Medical Intensive Care Unit (MICU) 2180-07-23 14:00:00 2180-07-23 23:50:47 0.4102662037037037*

*10000980 26913865 39765666 Medical Intensive Care Unit (MICU) Medical Intensive Care Unit (MICU) 2189-06-27 08:42:00 2189-06-27 20:38:27 0.4975347222222222*

*10001217 24597018 37067082 Surgical Intensive Care Unit (SICU) Surgical Intensive Care Unit (SICU) 2157-11-20 19:18:02 2157-11-21 22:08:00 1.1180324074074075*

**/*

*--------------------------------------------------------------------------------*

*Next speaker: sql-agent*

*sql-agent (to chat_manager):*

*```sql*

*-- Query to retrieve all basic information of ICU patients diagnosed with brain injury and older than 16 years*

*SELECT*

*p."subject_id",*

*p."gender",*

*p."anchor_age",*

*p."anchor_year",*

*p."anchor_year_group",*

*p."dod",*

*a."hadm_id",*

*a."admittime",*

*a."dischtime",*

*a."deathtime",*

*a."admission_type",*

*a."admit_provider_id",*

*a."admission_location",*

*a."discharge_location",*

*a."insurance",*

*a."language",*

*a."marital_status",*

*a."race",*

*a."edregtime",*

*a."edouttime",*

*a."hospital_expire_flag"*

*FROM*

*mimiciv_hosp.patients p*

*JOIN*

*mimiciv_hosp.admissions a ON p."subject_id" = a."subject_id"*

*JOIN*

*mimiciv_icu.icustays icu ON a."hadm_id" = icu."hadm_id"*

*JOIN*

*mimiciv_hosp.diagnoses_icd d ON a."hadm_id" = d."hadm_id"*

*JOIN*

*mimiciv_hosp.d_icd_diagnoses dicd ON d."icd_code" = dicd."icd_code" AND d."icd_version" = dicd."icd_version"*

*WHERE*

*dicd."long_title" ILIKE '%brain injury%' -- Assuming 'brain injury' is part of the diagnosis description*

*AND p."anchor_age" > 16;*

*```*

*This query retrieves all necessary patient and admission information for ICU patients diagnosed with brain injury and older than 16 years. It joins the relevant tables to filter based on the diagnosis description and age criteria.*

*--------------------------------------------------------------------------------*

*Next speaker: expert_agent*

*expert_agent (to chat_manager):*

*Your SQL query is well-structured and effectively retrieves the necessary information for ICU patients diagnosed with brain injury and older than 16 years. Here are a few points to consider for further improvement:*

*1. **Diagnosis Filtering**: The use of `ILIKE '%brain injury%'` is a good approach for case-insensitive matching. However, ensure that the term "brain injury" is consistently used in the `long_title` column for accurate filtering. If there are specific ICD codes for brain injuries, it might be more precise to filter using those codes.*

*2. **Column Selection**: You have selected a comprehensive set of columns from the `patients` and `admissions` tables. Ensure that all these columns are necessary for your analysis or reporting needs. If not, consider selecting only the essential columns to improve query performance.*

*3. **Formatting and Readability**: The query is well-formatted and easy to read, with clear separation of the `SELECT`, `FROM`, `JOIN`, and `WHERE` clauses. This enhances maintainability and understanding.*

*4. **Performance Considerations**: Ensure that the relevant columns used in joins and filters are indexed to optimize query performance, especially if the dataset is large.*

*Overall, your query adheres to PostgreSQL best practices and is structured effectively for the task at hand. BINGO!*

*--------------------------------------------------------------------------------*

*Next speaker: ICUagent*

(E) Example 3

If you want to extract the *subject_id of patients who underwent their first intubation within 72 hours of ICU admission. These patients must meet the following criteria: Age between 18 and 90 years. SOFA score of ≥10 on the first day of ICU admission.*

After requests input, ICU-GPT analyses and output the corresponding prompts as follows:

*ICUagent (to chat_manager):*

*When answering an input question:Query only the necessary columns from the relevant tables. Avoid querying all columns unless absolutely required. Wrap column names in double quotes (") to denote delimited identifiers. Ensure all queried columns exist in the provided tables.*

*Question: Identify the subject_id of patients who meet the following criteria for each ICU admission:*

*ICU Admission: The patient was admitted to the ICU (this includes all ICU admissions, not just the first).*

*Age: Between 18 and 90 years old.*

*SOFA Score: The patient had a SOFA (Sequential Organ Failure Assessment) score of ≥10 on the first day of ICU admission.*

*First Intubation: The patient underwent their first intubation within 72 hours of each ICU admission.*

*Only use the following tables:*

*CREATE TABLE mimiciv_derived.age (*

*subject_id INTEGER,*

*hadm_id INTEGER,*

*admittime TIMESTAMP WITHOUT TIME ZONE,*

*anchor_age SMALLINT,*

*anchor_year SMALLINT,*

*age NUMERIC*

*)*

*/**

*3 rows from age table:*

*subject_id hadm_id admittime anchor_age anchor_year age*

*10000084 23052089 2160-11-21 01:56:00 72 2160 72.89004154931676958169*

*10000084 29888819 2160-12-28 05:11:00 72 2160 72.99171500600210879971*

*10000764 27897940 2132-10-14 23:31:00 86 2132 86.78846315897709220492*

**/*

*CREATE TABLE mimiciv_derived.first_day_sofa (*

*subject_id INTEGER,*

*hadm_id INTEGER,*

*stay_id INTEGER,*

*sofa INTEGER,*

*respiration INTEGER,*

*coagulation INTEGER,*

*liver INTEGER,*

*cardiovascular INTEGER,*

*cns INTEGER,*

*renal INTEGER*

*)*

*/**

*3 rows from first_day_sofa table:*

*subject_id hadm_id stay_id sofa respiration coagulation liver cardiovascular cns renal*

*10546885 25213587 35947626 12 4 2 0 4 0 2*

*10321933 21721466 39606087 1 None 0 None 1 0 0*

*10371497 22500377 30409691 2 0 1 0 None None 1*

**/*

*CREATE TABLE mimiciv_derived.icustay_detail (*

*subject_id INTEGER,*

*hadm_id INTEGER,*

*stay_id INTEGER,*

*gender CHAR(1),*

*dod DATE,*

*admittime TIMESTAMP WITHOUT TIME ZONE,*

*dischtime TIMESTAMP WITHOUT TIME ZONE,*

*los_hospital NUMERIC,*

*admission_age NUMERIC,*

*race VARCHAR(80),*

*hospital_expire_flag SMALLINT,*

*hospstay_seq BIGINT,*

*first_hosp_stay BOOLEAN,*

*icu_intime TIMESTAMP WITHOUT TIME ZONE,*

*icu_outtime TIMESTAMP WITHOUT TIME ZONE,*

*los_icu NUMERIC,*

*icustay_seq BIGINT,*

*first_icu_stay BOOLEAN*

*)*

*/**

*3 rows from icustay_detail table:*

*subject_id hadm_id stay_id gender dod admittime dischtime los_hospital admission_age race hospital_expire_flag hospstay_seq first_hosp_stay icu_intime icu_outtime los_icu icustay_seq first_icu_stay*

*10000032 29079034 39553978 F 2180-09-09 2180-07-23 12:35:00 2180-07-25 17:55:00 2.2222222222222222 52.55996929585194352116 WHITE 0 1 True 2180-07-23 14:00:00 2180-07-23 23:50:47 0.41 1 True*

*10000980 26913865 39765666 F 2193-08-26 2189-06-27 07:38:00 2189-07-03 03:00:00 5.8069444444444444 76.4862311989189511 BLACK/AFRICAN AMERICAN 0 1 True 2189-06-27 08:42:00 2189-06-27 20:38:27 0.50 1 True*

*10001217 24597018 37067082 F None 2157-11-18 22:56:00 2157-11-25 18:00:00 6.7944444444444444 55.88148557820720386909 WHITE 0 1 True 2157-11-20 19:18:02 2157-11-21 22:08:00 1.12 1 True*

**/*

*CREATE TABLE mimiciv_derived.ventilator_setting (*

*subject_id INTEGER,*

*stay_id INTEGER,*

*charttime TIMESTAMP WITHOUT TIME ZONE,*

*respiratory_rate_set DOUBLE PRECISION,*

*respiratory_rate_total DOUBLE PRECISION,*

*respiratory_rate_spontaneous DOUBLE PRECISION,*

*minute_volume DOUBLE PRECISION,*

*tidal_volume_set DOUBLE PRECISION,*

*tidal_volume_observed DOUBLE PRECISION,*

*tidal_volume_spontaneous DOUBLE PRECISION,*

*plateau_pressure DOUBLE PRECISION,*

*peep DOUBLE PRECISION,*

*fio2 DOUBLE PRECISION,*

*flow_rate DOUBLE PRECISION,*

*ventilator_mode TEXT,*

*ventilator_mode_hamilton TEXT,*

*ventilator_type TEXT*

*)*

*/**

*3 rows from ventilator_setting table:*

*subject_id stay_id charttime respiratory_rate_set respiratory_rate_total respiratory_rate_spontaneous minute_volume tidal_volume_set tidal_volume_observed tidal_volume_spontaneous plateau_pressure peep fio2 flow_rate ventilator_mode ventilator_mode_hamilton ventilator_type*

*10007818 32359580 2146-07-04 05:00:00 None 23.0 23.0 608.0 None 340.0 300.0 None 5.0 40.0 None CPAP/PSV None Drager*

*10007818 32359580 2146-07-04 08:00:00 None 22.0 22.0 6.5 None 373.0 397.0 None 5.0 40.0 None CPAP/PSV None Drager*

*10007818 32359580 2146-07-04 12:00:00 None 29.0 29.0 8.4 None 377.0 386.0 None 5.0 40.0 None CPAP/PSV None Drager*

**/*

*--------------------------------------------------------------------------------*

*Next speaker: sql-agent*

*sql-agent (to chat_manager):*

*```sql*

*-- SQL Query to identify the subject_id of patients who meet the specified criteria for each ICU admission*

*SELECT DISTINCT icu."subject_id"*

*FROM mimiciv_derived.icustay_detail icu*

*JOIN mimiciv_derived.age age*

*ON icu."subject_id" = age."subject_id" AND icu."hadm_id" = age."hadm_id"*

*JOIN mimiciv_derived.first_day_sofa sofa*

*ON icu."subject_id" = sofa."subject_id" AND icu."hadm_id" = sofa."hadm_id" AND icu."stay_id" = sofa."stay_id"*

*JOIN (*

*SELECT "subject_id", "stay_id", MIN("charttime") AS first_intubation_time*

*FROM mimiciv_derived.ventilator_setting*

*WHERE "ventilator_type" IS NOT NULL*

*GROUP BY "subject_id", "stay_id"*

*) vent*

*ON icu."subject_id" = vent."subject_id" AND icu."stay_id" = vent."stay_id"*

*WHERE age."age" BETWEEN 18 AND 90*

*AND sofa."sofa" >= 10*

*AND vent.first_intubation_time <= icu."icu_intime" + INTERVAL '72 hours';*

*```*

*### Explanation:*

*1. **Tables Used**: The query uses the `icustay_detail`, `age`, `first_day_sofa`, and `ventilator_setting` tables.*

*2. **Joins**:*

*- The `icustay_detail` table is joined with the `age` table to filter patients based on age.*

*- It is also joined with the `first_day_sofa` table to filter based on the SOFA score.*

*- A subquery is used to find the first intubation time from the `ventilator_setting` table, which is then joined with the `icustay_detail` table.*

*3. **Conditions**:*

*- The patient's age is checked to be between 18 and 90.*

*- The SOFA score on the first day of ICU admission is checked to be 10 or more.*

*- The first intubation is checked to occur within 72 hours of ICU admission.*

*4. **Distinct**: The `DISTINCT` keyword ensures that each `subject_id` is returned only once, even if they have multiple ICU admissions meeting the criteria.*

*--------------------------------------------------------------------------------*

*Next speaker: expert_agent*

*expert_agent (to chat_manager):*

*The SQL query you provided is well-structured and adheres to PostgreSQL best practices. It accurately filters patients based on the specified criteria using appropriate joins and conditions. The use of `DISTINCT` ensures that each `subject_id` is returned only once, which is suitable for the requirement. The query is also well-formatted and easy to understand.*

*BINGO*

*--------------------------------------------------------------------------------*

*Next speaker: ICUagent*

(F) Example 4

If you want to extract *subject_id of patients aged 18 to 90 diagnosed with sepsis and receiving continuous renal replacement therapy within 24 hours of admission to the ICU.*

After requests input, ICU-GPT analyses and output the corresponding prompts as follows:

*ICUagent (to chat_manager):*

*When answering an input question:Query only the necessary columns from the relevant tables. Avoid querying all columns unless absolutely required. Wrap column names in double quotes (") to denote delimited identifiers. Ensure all queried columns exist in the provided tables.*

*Question:*

*Extract the subject_id of patients who meet the specified conditions, using disease name instead of ICD codes.*

*Conditions:*

*Diagnosed with Sepsis.*

*Age between 18 and 90 years.*

*Underwent Continuous Renal Replacement Therapy (CRRT) within 24 hours of ICU admission.*

*Approach:*

*Diagnosed with Sepsis: Use disease name or description (e.g., “Sepsis”).*

*Age filter: Between 18 and 90 years.*

*CRRT within 24 hours of ICU admission: Check the timing of CRRT relative to ICU admission.*

*Only use the following tables:*

*CREATE TABLE mimiciv_derived.age (*

*subject_id INTEGER,*

*hadm_id INTEGER,*

*admittime TIMESTAMP WITHOUT TIME ZONE,*

*anchor_age SMALLINT,*

*anchor_year SMALLINT,*

*age NUMERIC*

*)*

*/**

*3 rows from age table:*

*subject_id hadm_id admittime anchor_age anchor_year age*

*10000084 23052089 2160-11-21 01:56:00 72 2160 72.89004154931676958169*

*10000084 29888819 2160-12-28 05:11:00 72 2160 72.99171500600210879971*

*10000764 27897940 2132-10-14 23:31:00 86 2132 86.78846315897709220492*

**/*

*CREATE TABLE mimiciv_derived.crrt (*

*stay_id INTEGER,*

*charttime TIMESTAMP WITHOUT TIME ZONE,*

*crrt_mode TEXT,*

*access_pressure DOUBLE PRECISION,*

*blood_flow DOUBLE PRECISION,*

*citrate DOUBLE PRECISION,*

*current_goal DOUBLE PRECISION,*

*dialysate_fluid TEXT,*

*dialysate_rate DOUBLE PRECISION,*

*effluent_pressure DOUBLE PRECISION,*

*filter_pressure DOUBLE PRECISION,*

*heparin_concentration TEXT,*

*heparin_dose DOUBLE PRECISION,*

*hourly_patient_fluid_removal DOUBLE PRECISION,*

*prefilter_replacement_rate DOUBLE PRECISION,*

*postfilter_replacement_rate DOUBLE PRECISION,*

*replacement_fluid TEXT,*

*replacement_rate DOUBLE PRECISION,*

*return_pressure DOUBLE PRECISION,*

*ultrafiltrate_output DOUBLE PRECISION,*

*system_active INTEGER,*

*clots INTEGER,*

*clots_increasing INTEGER,*

*clotted INTEGER*

*)*

*/**

*3 rows from crrt table:*

*stay_id charttime crrt_mode access_pressure blood_flow citrate current_goal dialysate_fluid dialysate_rate effluent_pressure filter_pressure heparin_concentration heparin_dose hourly_patient_fluid_removal prefilter_replacement_rate postfilter_replacement_rate replacement_fluid replacement_rate return_pressure ultrafiltrate_output system_active clots clots_increasing clotted*

*30003226 2123-02-27 08:26:00 CVVHDF -44.0 120.0 180.0 0.0 Prismasate K2 1000.0 0.0 84.0 None None 0.0 1800.0 None Prismasate K2 200.0 35.0 None None None None None*

*30003226 2123-02-27 08:27:00 CVVHDF None None 1810.0 0.0 Prismasate K2 1000.0 None None None None None 1800.0 None Prismasate K2 200.0 None None 1 None None None*

*30003226 2123-02-27 09:00:00 None None None None None None None None None None None None None None None None None 230.0 None None None None*

**/*

*CREATE TABLE mimiciv_derived.icustay_detail (*

*subject_id INTEGER,*

*hadm_id INTEGER,*

*stay_id INTEGER,*

*gender CHAR(1),*

*dod DATE,*

*admittime TIMESTAMP WITHOUT TIME ZONE,*

*dischtime TIMESTAMP WITHOUT TIME ZONE,*

*los_hospital NUMERIC,*

*admission_age NUMERIC,*

*race VARCHAR(80),*

*hospital_expire_flag SMALLINT,*

*hospstay_seq BIGINT,*

*first_hosp_stay BOOLEAN,*

*icu_intime TIMESTAMP WITHOUT TIME ZONE,*

*icu_outtime TIMESTAMP WITHOUT TIME ZONE,*

*los_icu NUMERIC,*

*icustay_seq BIGINT,*

*first_icu_stay BOOLEAN*

*)*

*/**

*3 rows from icustay_detail table:*

*subject_id hadm_id stay_id gender dod admittime dischtime los_hospital admission_age race hospital_expire_flag hospstay_seq first_hosp_stay icu_intime icu_outtime los_icu icustay_seq first_icu_stay*

*10000032 29079034 39553978 F 2180-09-09 2180-07-23 12:35:00 2180-07-25 17:55:00 2.2222222222222222 52.55996929585194352116 WHITE 0 1 True 2180-07-23 14:00:00 2180-07-23 23:50:47 0.41 1 True*

*10000980 26913865 39765666 F 2193-08-26 2189-06-27 07:38:00 2189-07-03 03:00:00 5.8069444444444444 76.4862311989189511 BLACK/AFRICAN AMERICAN 0 1 True 2189-06-27 08:42:00 2189-06-27 20:38:27 0.50 1 True*

*10001217 24597018 37067082 F None 2157-11-18 22:56:00 2157-11-25 18:00:00 6.7944444444444444 55.88148557820720386909 WHITE 0 1 True 2157-11-20 19:18:02 2157-11-21 22:08:00 1.12 1 True*

**/*

*CREATE TABLE mimiciv_hosp.d_icd_diagnoses (*

*icd_code CHAR(7) NOT NULL,*

*icd_version SMALLINT NOT NULL,*

*long_title VARCHAR(255),*

*CONSTRAINT d_icd_diagnoses_pk PRIMARY KEY (icd_code, icd_version)*

*)*

*/**

*3 rows from d_icd_diagnoses table:*

*icd_code icd_version long_title*

*0010 9 Cholera due to vibrio cholerae*

*0011 9 Cholera due to vibrio cholerae el tor*

*0019 9 Cholera, unspecified*

**/*

*CREATE TABLE mimiciv_hosp.diagnoses_icd (*

*subject_id INTEGER NOT NULL,*

*hadm_id INTEGER NOT NULL,*

*seq_num INTEGER NOT NULL,*

*icd_code CHAR(7) NOT NULL,*

*icd_version SMALLINT NOT NULL,*

*CONSTRAINT diagnoses_icd_pk PRIMARY KEY (hadm_id, seq_num, icd_code, icd_version),*

*CONSTRAINT diagnoses_icd_admissions_fk FOREIGN KEY(hadm_id) REFERENCES mimiciv_hosp.admissions (hadm_id),*

*CONSTRAINT diagnoses_icd_patients_fk FOREIGN KEY(subject_id) REFERENCES mimiciv_hosp.patients (subject_id)*

*)*

*/**

*3 rows from diagnoses_icd table:*

*subject_id hadm_id seq_num icd_code icd_version*

*10000032 22595853 1 5723 9*

*10000032 22595853 2 78959 9*

*10000032 22595853 3 5715 9*

**/*

*--------------------------------------------------------------------------------*

*Next speaker: sql-agent*

*sql-agent (to chat_manager):*

*```sql*

*-- SQL Query to extract the subject_id of patients diagnosed with Sepsis,*

*-- aged between 18 and 90 years, and who underwent CRRT within 24 hours of ICU admission.*

*SELECT DISTINCT "icd"."subject_id"*

*FROM "mimiciv_hosp"."diagnoses_icd" AS "icd"*

*JOIN "mimiciv_hosp"."d_icd_diagnoses" AS "d_icd"*

*ON "icd"."icd_code" = "d_icd"."icd_code" AND "icd"."icd_version" = "d_icd"."icd_version"*

*JOIN "mimiciv_derived"."age" AS "age"*

*ON "icd"."subject_id" = "age"."subject_id" AND "icd"."hadm_id" = "age"."hadm_id"*

*JOIN "mimiciv_derived"."icustay_detail" AS "icu"*

*ON "icd"."subject_id" = "icu"."subject_id" AND "icd"."hadm_id" = "icu"."hadm_id"*

*JOIN "mimiciv_derived"."crrt" AS "crrt"*

*ON "icu"."stay_id" = "crrt"."stay_id"*

*WHERE "d_icd"."long_title" ILIKE '%sepsis%'*

*AND "age"."age" BETWEEN 18 AND 90*

*AND "crrt"."charttime" <= "icu"."icu_intime" + INTERVAL '24 hours';*

*```*

*This query performs the following steps:*

*1. Joins the `diagnoses_icd` table with the `d_icd_diagnoses` table to filter patients diagnosed with Sepsis using the disease name.*

*2. Joins with the `age` table to filter patients aged between 18 and 90 years.*

*3. Joins with the `icustay_detail` table to get ICU admission details.*

*4. Joins with the `crrt` table to ensure CRRT was performed within 24 hours of ICU admission.*

*5. Selects distinct `subject_id` values that meet all the specified conditions.*

*--------------------------------------------------------------------------------*

*Next speaker: expert_agent*

*expert_agent (to chat_manager):*

*The SQL query you've provided is well-structured and adheres to PostgreSQL best practices. It effectively joins the necessary tables to filter patients diagnosed with Sepsis, aged between 18 and 90 years, and who underwent CRRT within 24 hours of ICU admission. The use of `ILIKE` for case-insensitive matching of the disease name is appropriate, and the query correctly uses `DISTINCT` to ensure unique `subject_id` results.*

*BINGO*

*--------------------------------------------------------------------------------*

*Next speaker: ICUagent*
